# Sickness absenteeism among outsourced hygiene and cleaning workers at a university hospital in São Paulo, Brazil, 2015-2017

**DOI:** 10.5327/Z1679443520190450

**Published:** 2019-12-01

**Authors:** João Luiz Grandi, Mariana Cabrera Grell, Kelsy Catherina Nem Areco, Marcos Bossi Ferraz

**Affiliations:** 1 São Paulo Hospital, Universidade Federal de São Paulo - São Paulo (SP), Brazil. Universidade Federal de São Paulo São Paulo Hospital Universidade Federal de São Paulo Brazil; 2 Universidade Federal de São Paulo - São Paulo (SP), Brazil. Universidade Federal de São Paulo Universidade Federal de São Paulo Brazil

**Keywords:** occupational health, absenteeism, occupational diseases, housekeeping

## Abstract

**Background:**

Absenteeism refers to frequent absences from work without producing a medical certificate or having been granted sick leave.

**Objective:**

To establish the rate of short-term sickness absenteeism (1 to 15 days) among outsourced hygiene and cleaning workers at a university hospital.

**Methods:**

Retrospective analysis of all medical and dental certificates collected by the contractor along two years (2015 through 2017). Descriptive statistics analysis was performed according to the nature of variables (absolute and relative frequencies and measures of central tendency and variability for categorical and numerical variables respectively) followed by Pearson’s χ^2^ test.

**Results:**

The study population consisted of 370 workers, most of whom were female (n=333, 91.4%), aged 30 to 50 and with low educational level. The monthly absenteeism rate was 5.2%, on average, and the sickness absenteeism rate 37.9%. The most prevalent reasons for sick leave were musculoskeletal disorders (n=271, 20.7%) and diseases of the digestive system (n=141, 10.8%). The total number of missed work days along the analyzed period was 8,788.

**Conclusion:**

The epidemiological profile of sick leave spells included both occupational diseases (musculoskeletal disorders) and conditions without direct or indirect relationship to work. The proportion of sickness absence was low relative to the overall absenteeism rate.

## RESUMO |

**Introdução:**

Absenteísmo-doença é a ausência do trabalhador justificada por atestados médicos ou licença de saúde ao trabalho.

**Objetivo:**

Conhecer a frequência do absenteísmo-doença de curta duração (1 a 15 dias de afastamento), entre trabalhadores terceirizados do serviço de higiene e limpeza de um hospital universitário localizado na cidade de São Paulo.

**Métodos:**

Estudo de coleta retrospectiva de todos os atestados de saúde - médicos e odontológicos - recebidos pela empresa de limpeza contratada no período de dois anos (2015-2017). Os dados foram analisados por estatística descritiva de acordo com a natureza da variável (frequência absoluta e relativa para variáveis categóricas; medidas de tendência central e variabilidade para variáveis numéricas) e seguida do teste χ^2^ de Pearson.

**Resultados:**

Com 370 trabalhadores, a população do estudo foi caracterizada basicamente por mulheres (338; 91,4%) entre 30 e 50 anos de idade e de baixa escolaridade. A média de absenteísmo por mês foi de 5,2%, e a frequência do absenteísmo-doença, de 68,1%. As doenças mais prevalentes que causaram ao trabalhador o afastamento do trabalho foram as doenças osteomusculares (271; 20,7%) e as doenças do aparelho digestivo (141; 10,8%). O número de dias perdidos de trabalho foi de 8.788 no período do estudo.

**Conclusão:**

O perfil epidemiológico divide-se entre as doenças ocupacionais (doenças osteomusculares) e aquelas não relacionadas direta ou indiretamente às funções laborais. O percentual de dias perdidos por absenteísmo-doença foi considerado baixo, em proporção ao absenteísmo por todas as causas.

## INTRODUCTION

As an activity that endows structure and dignity to human life, work has a central role in the lives of people all around the world. It is through work that we define our identity and place in society^1^.

The labor market has undergone many changes over time, with considerable consequences for employment relationships. Hygiene and cleaning workers are one of the occupational groups that underwent a dramatic shift in their employment conditions - being these services now almost universally outsourced - however without significant implications for their lives^2^.

Workers in hospital hygiene services are charged of cleaning surfaces and the overall environment according to criteria of criticality and using chemicals and physical agents. According to the literature, this occupational group is predominantly composed of women with low educational level and income. Their tasks involve mainly repetitive movements and continuous physical effort^3^.

Cleaning tasks characteristically require the use of the hands, which increases the risk of several occupational diseases among which repetitive strain injury and work-related musculoskeletal disorders stand out^4^. Both conditions have high prevalence and strong relationship to work^3^^,^^4^^,^^5^. Recent studies performed with different occupational groups evidenced that mental and behavioral disorders^6^, musculoskeletal diseases and conditions due to external causes are the main causes of sickness among workers^7^. Spine and pelvic pain are the second most prevalent health problems in Brazil, following hypertension only^8^^,^^9^.

Hospital hygiene and cleaning is a complex activity which implementation and maintenance pose permanent challenges^4^. Continuous improvement and identifying factors associated with costs are necessary to ensure appropriate service delivery. One of such factors is absenteeism^2^.

Absenteeism is defined as unexpected absences from work^6^. As such, it causes serious management problems in organizations, since in addition to impacts on profit and productivity, it also hinders the continuity of service delivery.

Physical or mental health problems are one reason for such unplanned absences. This situation is known as sickness absenteeism and may be due to work-related or not diseases, occupational accidents or problems without any direct relationship to work.

Sickness absenteeism is influenced by countless factors and interferes with productivity, operational costs and the efficiency of work^7^. It is usually measured on the basis of medical certificates produced by absent workers and results from a complex interaction of individual, work and non-work related factors which increase costs, disorganize the work environment and impair productivity^6^^,^^7^^,^^8^.

Approaching such complex situation is difficult, especially in the case of outsourced cleaning workers, since few studies were performed in this regard^3^^,^^4^^,^^5^. Therefore, the aim of the present study was to analyze sickness absenteeism among cleaning workers at a high-complexity hospital in São Paulo, Brazil, in which these services are outsourced.

## METHODS

### STUDY DESIGN, SETTING AND PERIOD

The present is a retrospective, epidemiological, descriptive and analytical study of sickness absenteeism from September 2015 through December 2017 among outsourced cleaning workers at a philanthropic university hospital in São Paulo with national and international reputation in teaching and research. Cleaning services were outsourced in 2000, and the current agreement complies with the Ministry of Planning, Budget and Management Normative Instruction no. 2, from 30 April 2008, and later amendments.

### SAMPLE

The study sample was composed of all medical and dental certificates granting at least 1-day sick leave for conditions described in the International Classification of Diseases (ICD) 10 and kept by the contractor for payroll management.

### INCLUSION CRITERIA

We included all medical and dental certificates which indicated a health condition as reason for at least 1-day sick leave according to ICD-10 codes, the involved worker’s name, date, and doctor’s signature and stamp.

### EXCLUSION CRITERIA

For pedagogic and operational reasons we excluded all cases of sickness absence for which a medical certificate was not produced, as well as certificates in which a CID-10 code was not provided. We also excluded other reasons for missing work days, such school and/or judicial events and maternity/paternity leave, and certificates of work disability for more than 15 days issued by the National Social Security Institute - the latter because although incapacity for work was demonstrated it did not account for absences from work.

### STUDY PROTOCOL

Within 24 hours of returning to work, absentees were required to present a medical certificate to the contractor. The contractor’s nurse made copies of these certificates, delivered originals to the human resources department for payroll management and the copies to the outsourcer’s contract inspector.

Data extracted from medical certificates were entered on a Microsoft Excel spreadsheet tabulated according to the following variables: age, sex, position, work schedule, department, grade of insalubrity, basic monthly salary, duration of absence spells, dates, morbidity and diagnosis as per ICD-10 codes^10^. Relative to occupational variables (position, grade of insalubrity, department and work schedule) we considered those in vigor at the time medical certificates were issued regardless of eventual changes in position or previous benefits received.

To calculate the rate of absenteeism for the analyzed population, we considered events (sick leave spells) independently from the number of medical certificates presented by each involved worker. Thus we could establish the weight of events on the analyzed population and subcategories: different from the total number of involved population along a definite period, frequencies are not a measure of risk.

The absenteeism rate was calculated with the following equation:



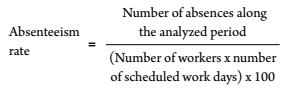
(1)


All effective employees at the time of data collection were invited to sign an informed consent form. Workers dismissed or having resigned more than 3 months earlier and those who had moved house or changed their telephone number were not invited to sign informed consent due to difficulties to locate them and eventual transportation expenses.

The principal investigator entered the collected data on an Excel spreadsheet. For the study purposes, sickness absenteeism was defined as absences from work due to disease or work accidents as established in medical or dental certificates issued by licensed professionals.

### STATISTICAL ANALYSIS

We performed exploratory analysis of the collected data and the results are presented in tables. Categorical variables were described as absolute and relative frequencies and the numerical variables as measures of central tendency (mean and median) and variability (minimum, maximum, standard deviation-SD, and interquartile range-IQR). Pearson’s X^2^ test was used to analyze associations between independent categorical variables. The significance level was set to 5%.

### ETHICAL ISSUES

The study project was uploaded to Platform Brazil and approved by the research ethics committee of Federal University of São Paulo on 10 August 2017, ruling no. 2380882, Certificate of Presentation for Ethical Appraisal no. 7381.13817.0000.5505.

The present study complies with the ethical principles described in the National Health Council Resolution no. 466/2012. All the participants were informed about the study aims and signed two copies of an informed consent form.

## RESULTS

The number of outsourced employees varied from 156 to 271, mean 243, along the analyzed period. The average duration of sickness absenteeism (with or without due justification) was 5.2 days, ranging from 1.29 to 16.8 (Table 1).

**Table 1.** Number of outsourced employees, absences (with or without medical certificate), absenteeism rate, number of medical certificates and number of granted sick leave days according to year and month, São Paulo, 2015-2017 (n=370).

Date		Employees	Absences	AR(%)	Without a medical certificate (N)	Without a medical certificate (%)	With a medical certificate (N)	Medical certificates (N)	Granted sick leave days (mean)
2015	Sept	271	250	3.54	241	96.4	9	8	1.1
	Oct	271	213	3.02	211	99.1	2	2	1
	Nov	271	207	2.93	166	80.2	41	28	1.5
	Dec	271	314	5.2	140	44.6	174	67	2.6
2016	Jan	263	260	4.34	124	47.7	136	62	2.2
	Feb	263	310	5.17	239	77.1	71	31	2.3
	Mar	263	317	4.63	258	81.4	59	21	2.8
	Apr	263	250	3.65	215	86	35	13	2.7
	May	263	266	3.89	260	97.7	6	3	2
	Jun	263	234	3.42	216	92.3	18	3	6
	Jul	263	229	3.34	189	82.5	40	6	6.7
	Aug	263	238	3.97	207	87	31	13	2.4
	Sept	263	201	3.35	82	40.8	119	44	2.7
	Oct	263	303	5.06	201	66.3	102	24	4.3
	Nov	263	302	5.04	117	38.7	185	127	1.5
	Dec	263	333	5.56	145	43.5	188	90	2.1
2017	Jan	263	320	5.35	94	29.4	226	73	3.1
	Feb	263	1.007	16.8	767	76.2	240	102	2.4
	Mar	263	616	10.3	422	68.5	194	83	2.3
	Apr	263	619	10.3	399	64.5	220	52	4.2
	May	263	718	11.9	636	88.6	82	47	1.7
	Jun	225	256	4.28	56	21.9	200	84	2.4
2017	Jul	225	341	5.69	194	56.9	147	75	2
	Aug	175	213	4.68	55	25.8	158	62	2.5
	Sept	156	117	2.88	58	49.6	59	59	1
	Oct	156	157	2.62	104	66.2	53	28	1.9
	Nov	156	120	2.9	22	18.3	98	63	1.6
	Dec	156	77	1.29	9	11.7	68	39	1.7
		243*	8,788	5.2*	5,827	68.1*	2,961	1,309	2.5*

AR: absenteeism rate; *mean.

A total of 8,788 absences by any reason was recorded relative to 370 workers, while medical certificates were presented for only 33.7% (n=2,961). The total number of certificates presented and analyzed was 1,309 and the average duration of sick leave spells 2.5 days (2,961/1,309). Sick leave spells of 10 or more days represented 2.5% (n=10) of the total. The mean number of medical certificates including an ICD-10 code was 3.54 per worker, mean 2 (IQR=3) and varying from 1 to 32 (data not shown).

As per the outsourcing contract terms and conditions, the proportion of male and female employees should be kept at around 15% and 75% respectively (data not shown). The rate of medical certificates granted to male employees tended to decrease, while that corresponding to female employees to increase; these results were statistically significant (p=0.027, linear trend test) (Table 2).

**Table 2.** Characteristics described in medical certificates per year, São Paulo, 2015-2017 (n=370).

Variables	2015		2016		2017		Total	
	N	%	N	%	N	%	N	%
Sex								
Male	10	9.5	29	6.6	36	4.7	75	5.7
Female	95	90.5	408	93.4	731	95.3	1.234	94.3
Total	105	100.0	437	100.0	767	100.0	1.309	100.0
Shift								
Morning	60	57.1	201	46.0	370	48.2	631	48.2
Afternoon	28	26.7	133	30.4	218	28.4	379	28.9
Night	3	2.9	41	9.4	80	10.4	124	9.5
Outpatient	14	13.3	62	14.2	99	13	175	13.4
Total	105	100.0	437	100.0	767	100.0	1.309	100.0
Grade of insalubrity								
Minimum (20%)	84	80.0	324	74.1	566	73.8	974	74.4
Maximum (40%)	21	20.0	113	25.9	201	26.2	335	25.6
Total	105	100.0	437	100.0	767	100.0	1.309	100.0
Basic salary	788.00		880.00		937.00		866.33*	

*mean.

The number of medical certificates increased for workers allocated to the night shift and decreased for those working the morning shift. However, this variation was not statistically significant (p=0.190, X^2^ test). Neither the difference in grade of insalubrity were significant (p=0.388).

Thirty-two (8.2%) employees were male and 338 (91.4%) female; this difference was statistically significant (p=0.001). Their average age was 38.17, median 37 (IQR=17) varying from 18 to 64 years old. About 60% of the analyzed medical certificates corresponded to employees aged 28 to 52 years old (data not shown) with small variation in frequency between those granted to workers under 20 (n=41, 3.1%) or aged 58 or older (n=47, 3.6%).

About 94.4% (n=1,235) of leaves were granted to female employees and only 5.6% (n=73) to males. The rate of medical certificates presented by women considerably increased in 2017 (55.8%). We did not find association between sex and analyzed year (p=0.0581).

The largest number of medical certificates were presented by employees allocated to the morning shift (n=631, 48.2%) followed by the afternoon shift (n=378, 28.9%). Absences of workers allocated to the night shift represented less than 10% of the total.

The largest proportion of absences (about 60%) corresponded to the warmest time of the year, 33% to the summer and 30.3% to the spring; the lowest rate corresponded to the autumn (14.8%).

As per the collective agreements approved by the Trade Union of Workers in Urban Cleaning and Conservation of São Paulo and as shown in Table 2, the basic salary of the analyzed occupational group increased from BRL 788.00 (2015) to BRL 937.00 (2017), mean BRL 868.33 along the analyzed period. With insalubrity additional, wages increased from 1,222.36 to BRL 1,453.15, mean BRL 1,300.95, median BRL 1.265.75, SD BRL 65.07 (BRL 1,222.35-1,453.33, IQR=3.67).

The largest proportion of absences corresponded to workers who received minimum-grade insalubrity additional (71.4%). Disease categories did not differ as a function of time of the year, except for respiratory disorders (p=0.003, Pearson’s X^2^) which were about twice more frequent in autumn/winter compared to spring/summer (Table 3).

**Table 3.** Diseases described in medical certificates according to year seasons, São Paulo, 2015-2017 (n=370).

Diseases	Spring/summer		Autumn/winter		Total	
	N	%	N	%	N	%
Other	185	22.0	107	22.8	292	22.3
Contact with health services; symptoms, signs and abnormal clinical and laboratory findings	184	21.9	98	20.9	282	21.5
Musculoskeletal system and connective tissue	183	21.8	88	18.8	271	20.7
Digestive system	87	10.4	54	11.5	141	10.8
Eye and adnexa	57	6.7	20	4.3	77	5.9
Respiratory system	54	6.4	61	13.0	115	8.8
Genitourinary system	45	5.4	23	4.9	68	5.2
Nervous system	45	5.4	18	3.8	63	4.8
Total	840	100.0	469	100.0	1.309	100.0

The most frequent reasons for absences were conditions included in the ICD-10 chapter XIII (diseases of the musculoskeletal system and connective tissue) to a total of 271 (20.71%) followed by chapter XI (diseases of the digestive system, n=191, 14.6%). Conditions corresponding to chapters III (diseases of the blood and blood-forming organs and certain disorders involving the immune mechanism), V (mental and behavioral disorders) and IV (endocrine, nutritional and metabolic diseases) represented less than 0.5% of reasons for sick leave described in medical certificates.

The low rates of seasonal and epidemic health problems is noteworthy, as e.g. those included in ICD-10 chapters VII (diseases of the eye and adnexa)-conjunctivitis 5.9%, VIII (diseases of the ear and mastoid process) 0.9%, and X (diseases of the respiratory system) 8.8%.

## DISCUSSION

In the present study we analyzed medical and dental certificates granting sick leave to outsourced employees of a highly reputed university hospital in which cleaning services were uninterruptedly outsourced from 2000 to 2018. These certificates were used to account for missed work days along two years (2015-2017).

A total of 5,827 out of a total of 8,788 missed work days were duly justified through presentation of medical certificates. The average annual absenteeism rate was 5.2%; sickness absence accounted for 68.1% of the total number of missed work days.

The largest proportion of medical certificates was granted to women. This finding was expected since for cultural reasons - recent changes notwithstanding - hygiene-related occupations are traditionally seen as female in Brazil^6^. Also other studies bear witness to the predominance of women in cleaning services^10^^,^^11^^,^^12^^,^^13^^,^^14^. In our study, this difference was statistically significant - 8.8% male vs. 91.4% female (p=0.0001) as also in the study by Chaves et al.^2^ who analyzed cleaning workers in northern Paraná, Brazil, and found that 66.6% were female. Similarly, Simões et al.^14^ found that proportion of female cleaning workers was 87.9%.

Despite recent technological advances, some occupations, such as cleaning, remain associated with a low educational level^6^. Our findings in this regard agree with those reported by Martins et al.^12^ relative to a public hospital, in which about 40% of the participants had attended elementary school only. In Hohenreuther et al.’s^3^ study, 1% of cleaning workers were illiterate and about 74% had 1 to 8 years of formal schooling. About 47% of outsourced cleaning workers in southern Brazil had not completed elementary school^10^.

About 60% of the analyzed workers were within the most economically productive age (28 to 52). This finding agrees with data reported for similar occupational groups; most participants in the studies by Hohenreuther et al.^3^ e Barros^15^ were aged 31 to 45 and 30 to 62, respectively.

Most sick leave spells had short duration, 1 to 3 days (66.8%). Similar data were reported by Hohenreuther et al.^3^ relative to 474 medical certificates issued to rural employees - including general and cleaning services - of a forestry company in Minas Gerais, Brazil, with 70% of certificates granting 1-day leave versus 30% for 15 days or longer. Differently, Santi et al.^10^ found that among cleaning workers in Curitiba, Brazil, most spells were short and requested to perform medical examinations - ICD-10 chapter XXI (contact with health services).

In a study performed at a public hospital in Belo Horizonte, Brazil, nurses were the occupational group with the highest rate of sick leave spells (43.7%) while among operational and outsourced workers this rate was 10.1%^7^. Almost half of rural workers under extremely adverse working conditions presented at least one medical certificate over one-year follow-up^3^.

In a study conducted with nursing staff at basic health care facilities in Campinas, Brazil, nurses were found to miss more work days than nursing technicians and assistants. This finding allows suggesting that as concerns sickness absence, nursing professionals with higher educational levels were absent from work longer by comparison to cleaning workers with lower salary^16^, the most common reasons being musculoskeletal disorders^1^.

To analyze the distribution of causes of sickness absenteeism we clustered the analyzed medical certificates according to ICD-10 chapters and codes. Several authors^9^^,^^11^^,^^17^^,^^18^ reported that musculoskeletal disorders are strongly associated to household chores and that that cleaning workers are the most prone to low back pain. Among hospital cleaning workers in northern Paraná, Brazil, who reported low back pain, it was related to job tasks such as lifting and carrying materials and heavy equipment as part of the everyday work routine^2^^,^^19^. Older studies found that the most recurrent conditions diagnosed by physicians to cleaning workers included cardiovascular (40.6%), gastrointestinal (21.7%) and musculoskeletal diseases (4.8%)^20^.

While in our study the rate of sick leave due to mental and behavioral disorders was just 0.2%, these conditions were the leading cause among nurses in Campinas, Brazil^16^. The authors of study performed with cleaning workers in southern Brazil reported an average of 2.24 (range: 1 to 15) certificates per worker, 31.8% of which corresponded to mental and behavioral disorders and 15.9% to musculoskeletal diseases^14^.

In several Brazilian hospitals hygiene workers are usually outsourced^21^. The overall aim of outsourcing is to reduce costs^22^, in addition to improving efficacy and the quality of products or services. However, sickness absenteeism has become a constant problem for both employers and employees. Sickness has negative consequences for people, but even more significantly for contractors, in as much as they must cover health expenses by themselves. Different from meal and transportation benefits and Outcome Participation Program contributions, contractors may not deduct the cost of health care.

Outsourced hospital workers are more exposed to all types of hazards - physical, chemical, mechanical, ergonomic and biological - than the rest of the staff^21^. By not being health care workers and as a function of outsourcing they belong to a different category of unionized workers. Within the ongoing competitive bidding system in Brazil, only 5% of service fees may be deduced due to absenteeism, which therefore makes it profitable for contractors, with the consequent impact on service delivery.

The present study, in which we analyzed a considerable number of medical certificates issued along two years, may represent the actual conditions and health care needs of outsourced employees. In addition, it may foster a revision of the criteria for public institutions to refuse paying missed days to contractors.

Despite institutional efforts to monitor, investigate and follow up absences of outsourced employees, the rate of medical certificates granting sick leave was low. Absences were daily recorded in the workplace by the outsourcing company, while medical certificates were gathered by the contractor and then delivered to the outsourcing company. For these reasons, more thorough research on determinants and risk factors of sickness absenteeism, its causes and frequency is needed. An additional limitation of the present study derives from the time frame set for data collection. Since we sought to analyze events based on seasonal variations, we also included the last quarter of 2015.

## CONCLUSION

The analyzed population was predominantly composed of female workers, of economically active age (28 to 52) and with low educational level. The number of missed work days was higher among the workers allocated to the morning shift and in the warmest time of the year. Main reasons for sickness absenteeism were musculoskeletal disorders (20.7%) followed by gastrointestinal diseases (10.8%). A noteworthy finding is the high frequency of sick leave spells attributed to ICD-10 chapter XXI, codes M00-M99-contact with health services for examination and investigation and no definite disease condition.

The rate of absenteeism due to sick leave as demonstrated by medical certificates with indication of a ICD-10 code was low. Diseases of the musculoskeletal system and connective system (CID-10 chapter XIII) were the most frequent in the analyzed medical certificates. Finally, we call the attention to the large number of medical certificates granting just 1-day leave.
